# Black dots in trichoscopy after COVID‐19. Can it be telogen effluvium?

**DOI:** 10.1111/dth.15053

**Published:** 2021-07-12

**Authors:** Maria Vastarella, Mariateresa Cantelli, Paola Nappa, Gabriella Fabbrocini, Sonia Sofía Ocampo‐Garza

**Affiliations:** ^1^ Dermatology Unit, Department of Clinical Medicine and Surgery University of Naples Federico II Naples Italy; ^2^ Departamento de Dermatología Universidad Autónoma de Nuevo León, Hospital Universitario ¨Dr. José Eleuterio González¨ Monterrey Nuevo León Mexico

To the editor,

With the growing number of COVID‐19 cases, more patients have experienced increased hair loss after SARS‐CoV‐2 infection.^1^ We report a case of a 58‐year‐old woman with atypical acute telogen effluvium (TE) after recovery from COVID‐19.

In February 2021, a patient came to our trichology outpatient clinic because of significant hair loss. The patient was diagnosed with COVID‐19 infection in November 2020 by performing a nasopharyngeal swab. The patient developed high fever and severe respiratory distress. She was hospitalized for 45 days and was treated with oxygen, azithromycin, prednisone, and enoxaparin. One month after the discharge she noticed severe diffuse hair loss, with trichodynia, especially in the vertex region. Trichologic examination revealed hair loss affecting the entire scalp with greater involvement of the vertex area (Figure 1(A)). A positive pull test was found. Trichoscopy in the frontal area, showed an increasing number of follicular units with one terminal hair, some yellow dots, and few upright regrowing hairs (Figure 1(B)). Meanwhile in the vertex area, a significant number of regrowing hairs (up to two or three in the same follicular unit), black dots and perifollicular discoloration were also found **(**Figure 1(C) **)**. Blood tests excluded vitamin B12, vitamin D, and iron deficiencies, autoimmune diseases, or thyroid dysfunction.

**FIGURE 1:**
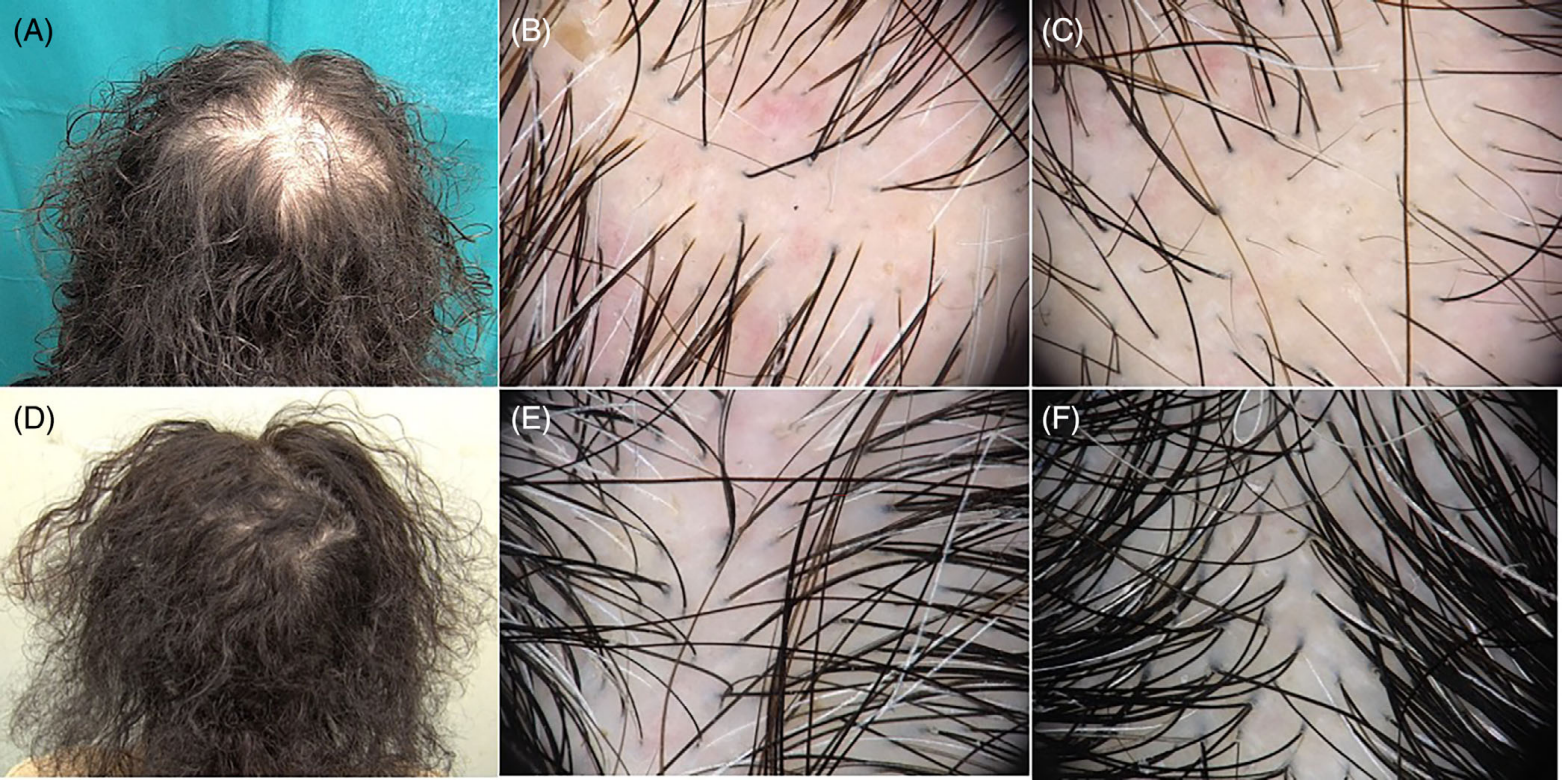
(A) Clinical image of patient with acute telogen effluvium and pressure alopecia. Severe diffuse hair loss, especially in the vertex region. (B) Dermoscopic image of the frontal region with an increased number of follicular units with one terminal hair, some yellow dots, and few upright regrowing hairs. (C) Dermoscopic image of the vertex region with several regrowing hairs (up to two or three in the same follicular unit), black dots, and perifollicular discoloration. (D) Clinical image of the patient after 2 months of treatment with clinical improvement. (E) Dermoscopic image of the frontal region after 2 months of treatment with presence of upregrowing hairs, no black dots or yellow dots were found. (F) Dermoscopic image of the vertex region after 2 months of treatment with presence of upregrowing hairs and improvement in hair density (Fotofinder ATM)

While the trichoscopic picture was compatible with TE in the frontal region, the vertex area showed black dots, typically not found in TE. After a direct anamnesis, the patient reported using a small pillow in the vertex area for head support during hospitalization. Therefore, we hypothesized a form of pressure‐induced alopecia (PA) in a patient with acute TE.

Treatment with oral minoxidil 0.5 mg/day, topical clobetasol once a day for a month and then twice a week until control, and a topical solution with caffeine and peptides once a week was started. After 3 months of treatment, the patient referred great improvement (Figure 1(D)). She denied hair shedding or trichodynia. The hair pull test was negative, and many upgrowing new hairs were found (Figure 1(E),(F)).

Recently, several cases of TE post‐SARS‐COV‐2 infection have been reported in the literature which appear to be related to the “cytokine storm” present mainly in patients with severe disease.^2^ We present a new case of COVID induced alopecia: pressure alopecia with concomitant TE. PA is a term used to describe a group of scarring and nonscarring alopecias that often occur following hospitalization in intensive care units.^3^

Hair loss happens because of tissue ischemia caused by prolonged pressure on the scalp, which results in compression of the vessels surrounding the hair follicles with the cessation of their activity. Hypoxia may be amplified by the activation of the coagulation cascade and decreased anticoagulant proteins induced by SARS‐COV‐2 infection. These factors may lead to the formation of a microthrombi, which may occlude the blood supply to the hair follicle.^4^ In our case an increase in the D‐dimer and fibrinogen was found, which could in part explain the pathogenesis. Trichoscopic features include black dots, broken hairs, erythema, and ulceration.^4^ Circle hairs are usually a favorable prognostic finding of reversible PA.^5^ Identifying the correct type of post COVID‐19 alopecia must be a prerogative of the following studies to allow early identification and establish the correct therapy. Preventive measures in intensive care units, including intermittent head repositioning and scalp massage, should be considered to reduce the incidence of PA.

## CONFLICT OF INTEREST

None declared.

## Data Availability

The data that support the findings of this study are available from the corresponding author upon reasonable request.
